# Cognitive Radio Networks for Internet of Things and Wireless Sensor Networks

**DOI:** 10.3390/s20185288

**Published:** 2020-09-16

**Authors:** Heejung Yu, Yousaf Bin Zikria

**Affiliations:** 1 Department of Electronics and Information Engineering, Korea University, Sejong 30019, Korea; heejungyu@korea.ac.kr; 2 Department of Information and Communication Engineering, Yeungnam University, 280 Daehak-Ro, Gyeongsangbuk-do, Gyeongsan-si 38541, Korea

**Keywords:** CRN, IoT, WSN, 5G, spectrum sensing, spectrum sharing

## Abstract

Recent innovation, growth, and deployment of internet of things (IoT) networks are changing the daily life of people. 5G networks are widely deployed around the world, and they are important for continuous growth of IoT. The next generation cellular networks and wireless sensor networks (WSN) make the road to the target of the next generation IoT networks. The challenges of the next generation IoT networks remain in reducing the overall network latency and increasing throughput without sacrificing reliability. One feasible alternative is coexistence of networks operating on different frequencies. However, data bandwidth support and spectrum availability are the major challenges. Therefore, cognitive radio networks (CRN) are the best available technology to cater to all these challenges for the co-existence of IoT, WSN, 5G, and beyond-5G networks.

## 1. Introduction

The numerous Internet of Things (IoT) technologies have contributed to explosive increase in the number of Internet-connected devices. The exponential growth of these *smart* connected devices is driving the revolution in social life of people. Move-forward in such a direction is the fifth-generation (5G) and beyond-5G connectivity. The IoT driven by 5G is shaping the future of smart connectivity in the society [1]. For different IoT services, the quality of service (QoS) is evaluated by parameters, such as latency, reliability, power consumption as well as throughput. In addition, its computing capabilities, memory, and energy efficiency determine the performance of the connected devices. The deployment of cognitive radio network (CRN) techniques for future wireless networks could be helpful in realizing the envisioned tactile Internet [2], sustainable information centric network [3,4], and intelligent next generation networks [5,6].

The rest of the editorial is organized as follows. Section 2 provides future research directions. Section 3 summarizes the accepted papers. Finally, Section 4 concludes the editorial.

## 2. 5G and Beyond-5G IoT Based on Cognitive Radio Networks

The limited availability of the communication spectrum is one of the challenges which hinder the massive deployment of the 5G and beyond-5G based IoT systems. The huge demand for the spectrum raises the difficulties in allocating the available frequency band. At the same time, due to various reasons, a large range of frequency band remain under-utilized. The 5G and beyond-5G communication networks are expected to leverage the under-utilized frequency band as well as increase the bandwidth by resolving the issue of spectrum scarcity for the billions of anticipated devices connected the Internet. The 5G and beyond-5G are intended to support the ultra-dense radio access networks (UDRANETs) that will accommodate a vast number of devices operating in millimeter wave (mmWave) and THz frequency, which are currently unregulated except for certain proprietary cellular networks. Spectrum sharing is one of the strategies used to alleviate such difficulties. The concept of spectrum sharing is basically for multiple networks to use the spectrum in respect of a given temporal and spatial configuration. CRNs realize such spectrum sharing and effectively use the available bands, i.e., under-utilized spectrum. The cognitive radios (CRs), which are built on the principle of software-defined radios (SDRs), are capable of intelligent context-aware sharing. The goal of the CRs in 5G and beyond-5G is to alleviate spectrum crunch as well as spectrum under-utilization. The CR has two capabilities as follows:

- *Cognitive ability*: The capability to sense the surrounding spectrum environments to identify the available frequency, i.e., under-utilized spectrum. The CR will decide whether the particular band is idle or occupied at a given time and location.
- *Re-configurability*: This capability allows the radio to dynamically program the system in order to communicate with another set of frequencies and access technologies specified by the underlying hardware.

The CRN allows dynamic spectrum access and can reconfigure the properties of spectrum utilization. In CRN topologies, there are two groups of users, grouped according to the priority of accessing the network. The principal users are called primary users (PUs), while the opportunistic users are called secondary users (SUs). The PUs have the priority by paying for band allocation, while the SUs are temporary spectrum consumers when the frequency is not used by the PU. The sharing of the spectrum between these two classes is the primary goal of the CRN while ensuring the performance for both the classes is maintained. One of the major tasks in this operation is to ensure that PU’s priority is secured, while at the same time retaining the QoS for the SUs. To ensure a QoS-compliant efficiency, moreover, the interference between the PUs and the SUs should be minimized. Thus, the CRNs allow for the coexistence of unlicensed users with the licensed users amid challenges such as user intervention and optimization of the power and resource allocation of the SUs.

Two major architectures, i.e., centralized and decentralized architectures, are adopted in the CRNs, based on the implementation of the IoT framework. A cooperative sharing of spectrum can be followed at the centralized CRN. Various cognitive radio units (CRUs) can be clustered together by adding a cluster head to reduce the overhead signal and computational expense. This method allows cooperative use of knowledge from the CRUs in the cluster. For non-cooperative sharing of bandwidth, on the other hand, the CRUs follow a greedy approach to maximize the individual capacity. This approach does not use the interference information from the other CRUs. In addition, the total ability of the network and the efficiency of the spectrum are adversely affected. There are four types of access methodology for the sharing of spectrum, namely interweave spectrum sharing, underlay spectrum sharing, overlay spectrum sharing, adaptive spectrum sharing. Each technique is employed according to the QoS requirements of IoT applications. These strategies vary in the way of the SUs’ channel access in relation to the PUs. The steps involved in the CR process present many challenges in network design. The first step is to collect spectral, geographic, and topological details. The status of the network resources is then determined along with the network protocols and policies. The required communication technique is implemented, based on this knowledge.

There are various problems facing the CRNs in terms of both network architecture and hardware. For IoT terminals, the required features include interoperability with various technologies, such as radio environment context awareness, communication pattern learning intelligence, low-power self-optimization, and spectrum usage robustness. The CR networks are needed to be effective both in terms of spectrum utilization and network capacity. There are also challenges in terms of interoperability and co-existence between multiple communication standards. The support for various techniques should be provided for seamless operation of many connected CRUs. The CRNs should be able to accommodate a large amount of data generated by a large number of devices in real-time, i.e., with low latency. To boost network performance, therefore, a CRN cloud architecture has been implemented. However, this raises a new set of research challenges. Future research areas are listed as follow:

- Interoperability challenges
  - CRN based device-to-device communications
  - CRN based device-to-network communications
  - CRN based machine-to-machine communications
  - CRN based service-to-service communications
- Co-existence network technologies challenges
  - CRN based radio access
  - CRN based medium access control (MAC)
  - CRN based handover between multiple communication standards
  - CRN based channel hopping
- Resource management challenges
  - CRN based resource allocation for the heterogenous CRUs
  - CRN based radio resource management (RRM) for spectrum sensing and interference avoidance
  - CRN based dynamic RRM systems for centralized and non-centralized sharing
- Intelligent CRN challenges
  - Machine learning (ML)/ deep learning (DL)/ reinforcement learning (RL) based MAC for CRN
  - ML/DL/RL based routing protocols for CRN
  - ML/DL/RL based Handover between the CR Base Stations
  - ML/DL/RL based offloading in the CR enabled cloud services
  - ML/DL/RL based spectrum sensing and spectrum sharing strategies
- CRN based cloud services challenges
  - Offloading techniques
  - Co-operative sensing
  - Geo-location identification
- CRN based hardware challenges
  - Interoperability between multiple radios
  - Antenna design for massive multiple-input multiple-output (MIMO)
  - Solutions for mmWave and THz channels and miniaturization of radios
- CRN based energy efficient approaches
  - Low power CR node hardware design
  - Duty-cycling for SUs
  - Low power spectrum sensing
- Security and Privacy challenges for CRN

## 3. A Brief Review of Articles in Special Issue

The massive deployment of IoT devices requires additional spectrum resources to provide guaranteed QoS of future wireless networks. However, a major obstacle to resolve this problem is spectrum depletion. Many techniques are employed to reuse or share the spectrum among multiple users without degrading the system performance. Miah et al. [7] proposed an enhanced spectrum sharing technique by utilizing kullback leibler divergence (KLD). Their approach shows a reasonable sensing performance even with the limited sample for sensing and noise uncertainty. They provided mathematical analysis and experiments to show the effectiveness of the proposed scheme. It is shown that the proposed sensing method can achieve the improved probability of detection and higher sum rate compared to the conventional techniques.

Satellite communication networks provide extensive comprehensive coverage to the remotely deployed devices. A digital channelizer is used to provide support for multiple transponders in satellite communications. Kim et al. [8] proposed an energy monitoring dependent cognitive communication system for detecting and cancelling interference. They used the inherent properties of the digital channelizer and fast fourier transform (FFT) to improve interference detection performance. They evaluated the analytical sensing performance mathematically, and used the empirical results to verify it. The results showed that the proposed method tackled tone interference detection and cancellation effectively.

Technology innovation to make devices energy efficient is accelerating at an unprecedented pace. It is also ideal for wireless devices with limited power to increase the device lifetime and, consequently, network lifetime [9]. In case of network bottleneck, wireless sensor networks (WSN) can use CRN technology to increase throughput. The leasing of PU’s spectrum to SU is one of the viable options to increase the spectrum reusability and profit for service providers. Xu et al. [10] proposed a differential game model for energy efficient allocation of cognitive WSN resources. By using open loop Nash equilibrium and feedback Nash equilibrium, the authors formulated optimal game control strategies for SU. They evaluated the proposed game technique with numerical simulations. The results show that the optimal solutions for cognitive WSN users are available to ensure the accuracy and efficacy of the proposed scheme.

In the literature, the relaying wireless sensor nodes are used to efficiently transmit the data to the destination. It increases the transmission capacity of the network, and saves energy. The network consists of secondary source (SS) node, secondary relay (SR) nodes, secondary destination (SD) nodes, and primary destination (PD) nodes. Lee et al. [11] proposed a cooperative phase steering (CPS) technique with duty cycling to maximize spectrum sharing and boost energy efficiency. They evaluated the CPS mathematically first, and then tested the scheme using comprehensive mathematical simulations. The results show the improved efficiency as compared with the traditional relay selection scheme in terms of throughput and outage probability.

The application of unmanned aerial vehicles (UAV) is growing in smart cities and IoT. The concern about reliability and security is especially essential for ensuring protection and preventing any accidents. Energy harvested UAV communications are essential to prolonging battery life. CR based communication for UAV may be useful by utilizing already available spectrum without dedicated channel and increase coverage. Khalid et al. [12] proposed UAV energy management scheme based on CR technology. They developed an analytical model for the connection outage probability, secrecy outage probability, and residual energy. To minimize the energy consumption, they devised optimal sensing duration and transmission power. They also considered the scenario for the eavesdroppers to illustrate the security and reliability of the proposed scheme. The results guarantee the secrecy and reliability of the proposed system with energy efficiency.

Optimizing network resources is important for achieving spectrum efficiency among CR IoT. When taking into account multiple parameters simultaneously such as transmission power, delay, and transmission rate, it becomes a very complex problem to solve. Muwonge et al. [13] proposed an optimal solution in the presence of a bit error rate (BER) and interference, by considering all these factors together. They investigated how total power, rate and delay vary depending on the packet size, network size, BER and interference. They used the branch-and-cut polyhedral method to solve this problem. They observed that a larger packet size and more number of SUs lead to increase power, interference, and transmission delay. As a result, it affects the BER performance and, eventually, the transmission rate. It is interesting to evaluate the proposed scheme with the related ones to see the overall system improvement effectively.

## 4. Conclusions

Six papers in this SI present state-of-the-art research developments in the field of Cognitive Radio Networks for Internet of Things and Wireless Sensor Networks. The papers gave the readers insight and innovative ideas of CRNs. The guest editors would like to express gratitude to the authors and thank all the anonymous reviewers for providing positive feedback to enhance the overall content of all the papers they have accepted. We would also like to thank editor-in-chief Prof. Dr. Vittorio M.N. Passaro, Prof. Dr. Leonhard M. Reindl, Prof. Dr. Assefa M. Melesse, Prof. Dr. Alexander Star and managing editor Angelina Wang for the invaluable help and productive advice in finalizing this SI.

## Abbreviations

The following abbreviations are used in this manuscript:

5G	Fifth-Generation
BER	Bit Error Rate
CPS	cooperative Phase Steering
CRN	Cognitive Radio Network
CRs	Cognitive Radios
CRUs	Cognitive Radio Units
FFT	Fast Fourier Transform
IoT	Internet of Things
PUs	Primary Users
QoS	Quality of Service
SDRs	Software-Defined Radios
SUs	Secondary Users
UAV	Unammaned Aerial Vehicles
UDRANETs	Ultra-Dense Radio Access Networks
WSN	Wireless Sensor Networks
